# Fast fringe-field switching of a liquid crystal cell by two-dimensional confinement with virtual walls

**DOI:** 10.1038/srep27936

**Published:** 2016-06-15

**Authors:** Tae-Hoon Choi, Seung-Won Oh, Young-Jin Park, Yeongyu Choi, Tae-Hoon Yoon

**Affiliations:** 1 Pusan National University, Department of Electronics Engineering, Busan, 46241, Korea

## Abstract

We report a simple method for reducing the response time of a fringe-field switching liquid crystal cell by using two-dimensional confinement of the liquid crystals. Through both numerical calculations and experiments, we show that the switching speed can be increased by several fold in a fringe-field switching cell by simply using a rubbing angle of zero, which causes virtual walls to be built when an electric field is applied between the interdigitated electrodes and the common electrode, without requiring additional fabrication steps or complicated drive schemes. Furthermore, the devices fabricated with this method exhibit a reduced color shift and excellent dynamic stability, even with a high applied voltage and under external pressure.

Over the past few decades, liquid crystal display (LCD) technologies have been rapidly developed because of strong competition among various technologies. As a result, LCDs now play a leading role in flat-panel display devices. Several liquid crystal (LC) modes have been successfully commercialized, such as the twisted nematic^1^, vertical alignment^2^, in-plane switching (IPS)^3^, and fringe-field switching (FFS)^4^,^5^ modes. Among these, the IPS and FFS modes exhibit superior performance in terms of view angle, color shift, and pressure resistance for touch panels. In particular, the FFS mode has been widely used in mobile displays because of its outstanding features, such as a low operating voltage and high transmittance.

However, the response time of FFS cells is still relatively long because the restoring elastic torque is primarily governed by the twist elastic constant *K*_22_, which is generally smaller than the splay elastic constant *K*_11_ and the bend elastic constant *K*_33_. The negative effects of a long response time include motion blur and deteriorated image quality. For these reasons, overcoming the slow response remains an important technical issue to be solved. Recently, rapid growth of the digital signage and vehicular display markets has caused short response times to become an essential requirement for LCD devices to operate well under all possible ambient conditions, especially in low temperature environments. To reduce the response time of an FFS cell, various methods have been proposed^6^,^7^,^8^,^9^,^10^,^11^,^12^,^13^,^14^,^15^,^16^. However, these methods all have drawbacks, such as complicated drive schemes, low transmittance, high operating voltage, and low image quality resulting from a non-uniform distribution of the residual polymer materials.

In this paper, we report a simple method for achieving a short response time in an FFS cell by using two-dimensional confinement of the liquid crystals. Through both numerical calculations and experiments, we show that the switching speed can be increased by several fold in an FFS cell by simply using a rubbing angle of zero, which causes virtual walls to be built when an electric field is applied between the interdigitated electrodes and the common electrode, without requiring additional fabrication steps or complicated drive schemes.

## Principle

**Figure 1:**
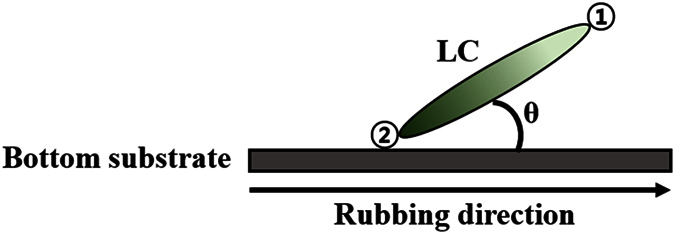
LC molecule tilted by rubbing of the alignment layer on the bottom substrate in a homogeneously aligned LC cell.

**Figure 2:**
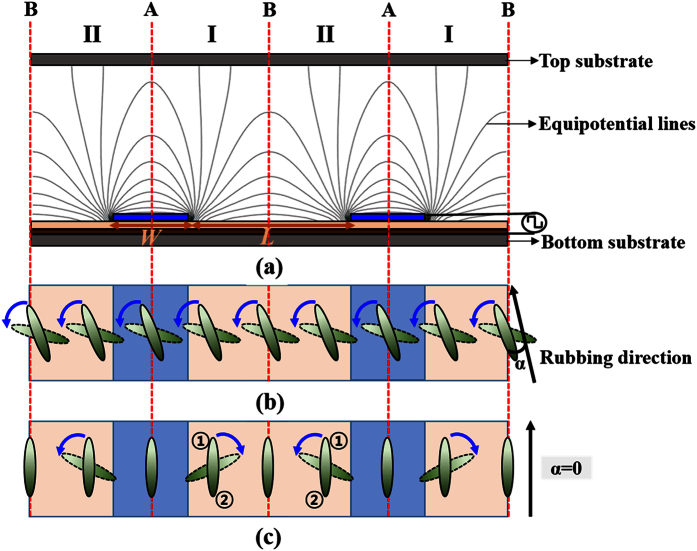
FFS cell structures. (**a**) Cross-sectional view with equipotential lines. Top view with (**b**) rubbing angle α ≠ 0 and (**c**) α = 0.

FFS cells require a rubbing process to homogeneously align the nematic LCs. As a result of the surface rubbing, the LC director has a small tilt angle θ, as shown in Fig. 1. The rubbing angle α is typically chosen to be between 5° and 15° with respect to the interdigitated electrodes in FFS cells that use LCs with positive dielectric anisotropy, as shown in Fig. 2(b). The transmission axis of the bottom polarizer is oriented along the rubbing direction of the bottom substrate, and the top and bottom polarizers are perpendicular to each other. In the absence of an applied electric field, the alignment direction of the LC molecules coincides with the transmission axis of the polarizer, and the cell is in the dark state. When a voltage higher than the Freedericksz transition threshold is applied to the cell, as shown in Fig. 2(a), the LC molecules are rotated in the direction of the applied fringe field, and the cell switches to the bright state, as shown in Fig. 2(b). The direction of rotation of the LC molecules in a conventional FFS cell with α ≠ 0 is independent of the pretilt direction.

In contrast, when a voltage is applied to an LC cell with rubbing angle α = 0, the direction in which the LC molecules rotate is indeterminate if the pretilt angle θ is zero, because the direction of the applied fringe field is always perpendicular to that of the LC director. However, if the pretilt angle θ is non-zero, the fringe field applied to the LCs breaks the symmetry of the rotational torque in the LC molecules. The tilted ends of the LC molecules, represented as point ① in Figs 1 and 2(c), are rotated along the direction of the applied fringe field, because the anchoring energy at point ① is lower than that at point ②.In other words, the direction in which the LCs rotate is determined by the pretilt direction of the LC molecules. Because the direction of the in-plane component of the applied fringe field in region I is opposite to that in region II, as shown in Fig. 2(a), the LC molecules in region I are rotated in the clockwise direction, but those in region II are rotated in the counter-clockwise direction, as shown in Fig. 2(c). The LC molecules at boundaries A and B, represented by dotted red lines in Fig. 2(a), are not rotated, as shown in Fig. 2(c), because there are no in-plane components in the applied electric field.

## Results and Discussion

### Calculated results

To confirm the dynamic switching behavior of the LCs, the Ericksen-Leslie equation coupled with the Laplace equation was numerically solved using the finite-element method, which is generally used to describe the motion of LC molecules. We performed numerical calculations using the TechWiz LCD 2D (Sanayi System Company, Ltd., Korea) software package. The parameters of the LC material used in the numerical calculations were as follows: the elastic constants *K*_11_ = 11.3 pN, *K*_22_ = 5.9 pN, *K*_33_ = 14 pN, the optical anisotropy ∆*n* = 0.119, the dielectric anisotropy ∆ɛ = 5.1, and the rotational viscosity γ_1_ = 47.6 mPa·s. The cell gap *d* was 3.5 μm, and the pretilt angle was 2°. The width *W* of the interdigitated electrodes and the gap *L* between them were 2.8 μm and 6 μm, respectively.

**Figure 3:**
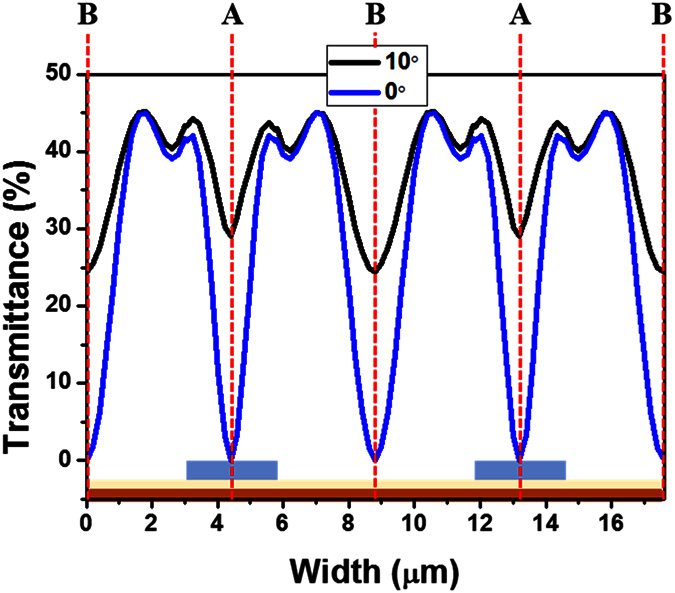
Calculated transmittance distribution of an FFS cell.

**Figure 4:**
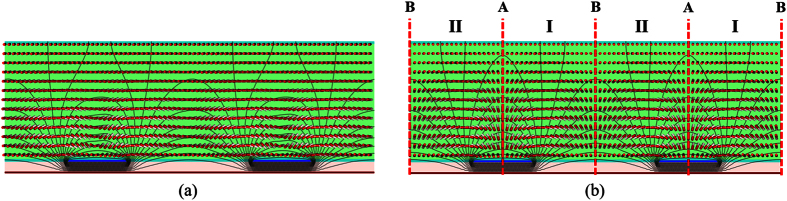
LC director orientation and equipotential line in an FFS cell. (**a**) α = 10°, (**b**) α = 0°.

Figure 3 shows the calculated transmittance distribution of the FFS cells. The LC director distributions and the equipotential lines in the FFS cells are shown in Fig. 4. In an FFS cell with rubbing angle α ≠ 0, all of the LC molecules are rotated in the same direction, which results in a twist deformation of the LC molecules, as shown in Fig. 4(a). Although the transmittance distribution is not uniform, the incident light is transmitted over the entire cell area, as shown in Fig. 3. In contrast, in an LC cell with α = 0, the LC molecules in region II are rotated in the direction opposite to those in region I when a fringe field is applied to the LC cell, as shown in Fig. 4(b). At the boundaries between regions I and II, there is no change in the azimuth angle of the LC director because there are no in-plane components of the applied electric field. As a result, no light is transmitted at boundaries A and B, as shown in Fig. 3. Although there is no change in the azimuth angle, the LC molecules at boundaries A and B are tilted when a fringe electric field is applied to an LC cell with α = 0, which may result in a longer turn-off time.

**Figure 5:**
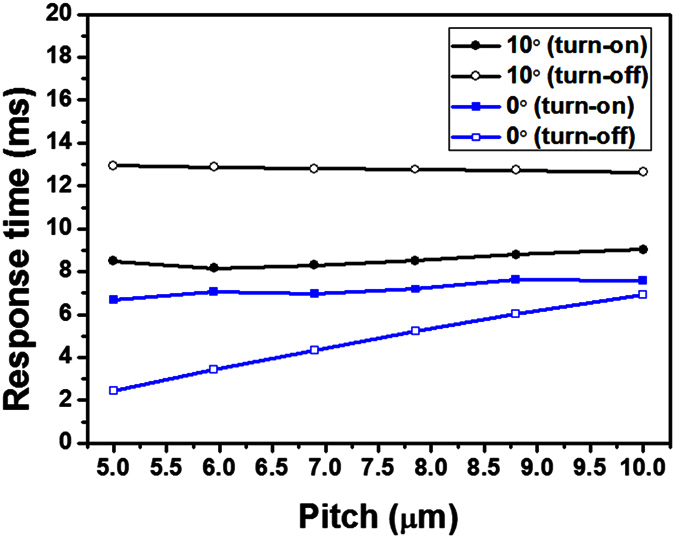
Dependence of the response time on the pitch of interdigitated electrodes in an FFS cell.

**Figure 6:**
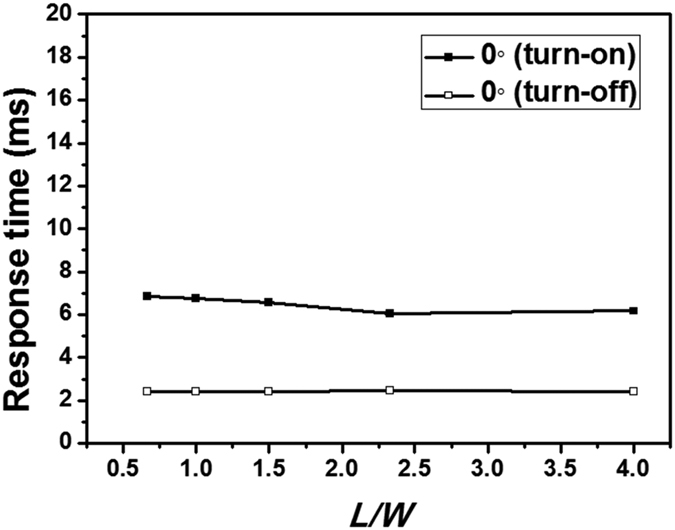
Response time of an FFS cell with α = 0 vs. the ratio *L*/*W* for a fixed pitch of 5 μm in the interdigitated electrodes.

We also calculated the response time of an FFS cell as the pitch of the interdigitated electrodes was varied, as shown in Fig. 5. Here, we defined the turn-on [turn-off] time as the transient time from 10% [90%] to 90% [10%] of the maximum transmittance. In an FFS cell with α ≠ 0, the response time is independent of the pitch of the interdigitated electrodes. In contrast, in an FFS cell with α = 0, the response time is largely dependent on the pitch of the interdigitated electrodes. In the FFS cells with α = 0, the turn-on [turn-off] time decreased from 7.61 ms [6.92 ms] to 6.68 ms [2.43 ms] when the pitch was reduced from 10 μm to 5 μm, respectively. When the pitch was 5 μm, the turn-off time was five times shorter than that of an FFS cell with α = 10°. The response time was not significantly affected by the ratio of the width of the interdigitated electrodes and the gap between them when the pitch was maintained at a fixed value, as shown in Fig. 6. Hence, the pitch of the interdigitated electrodes is a dominant factor that affects the response time of an FFS cell with α = 0.

As shown in Fig. 5, FFS cells with α = 0 showed much faster turn-on switching than FFS cells with α ≠ 0, regardless of the pitch of the interdigitated electrodes. In an FFS cell with α ≠ 0, LC molecules near the interdigitated electrode edge, where the in-plane component of the applied electric field is very high, are rapidly rotated, whereas the LC molecules at A and B, where the applied electric field has no in-plane component, are rotated only by the elastic torque, thus resulting in slow turn-on switching. In contrast, in an FFS cell with α = 0, the LC molecules in region II are rotated in the direction opposite to those in region I when an electric field is applied to the FFS cell. During this turn-on switching process, the LC molecules at boundaries A and B between regions I and II are not rotated by the elastic torque; hence, the turn-on switching of an FFS cell with α = 0 is primarily governed by the dielectric torque, resulting in fast turn-on switching. Moreover, the elastic energy is very high at the domain boundaries A and B because the LC molecules are not rotated by the elastic torque, in contrast to an FFS cell with α ≠ 0, resulting in faster turn-off switching of an FFS cell with α = 0. Boundaries A and B between regions I and II can be considered to be virtual walls. The LC molecules in an FFS cell with α = 0 are confined not only by the two substrates but also by these virtual walls, and thereby a short response time can be achieved.

**Figure 7:**
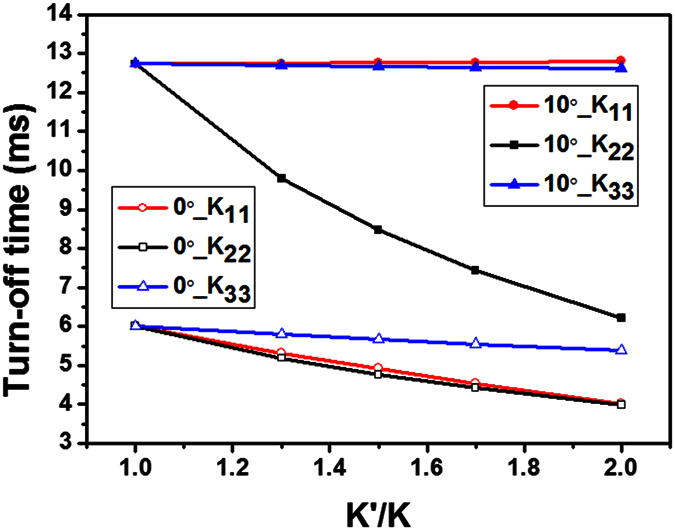
Dependence of the turn-off time on three elastic constants (*W*: *L* = 2.8 μm: 6 μm).

To investigate the dependence of the response time of FFS cells on the elastic constants, we calculated the response time by varying each elastic constant while holding all other material parameters constant. Figure 7 shows the dependence of the turn-off time on each elastic constant in an FFS cell. In an FFS cell with α = 10°, the turn-off time remained unchanged as *K*_11_ and *K*_33_ were varied, indicating that the turn-off time was independent of *K*_11_ and *K*_33_. In contrast, in an FFS cell with α = 0, the turn-off time was dependent on both *K*_11_ and *K*_22_. In other words, both splay and twist deformation occurred when a fringe electric field was applied to an FFS cell with α = 0. The field-induced splay deformation contributed to a reduction in the response time of the FFS cell with α = 0. This result confirms the two-dimensional confinement of the LCs by virtual walls.

The calculated results presented in this paper were obtained by assuming a pretilt angle θ of 2°, which is a typical value for the conventional rubbing method. The LCs in an FFS cell with a lower pretilt angle start to switch more slowly in the initial stages. However, the pretilt angle does not have a significant effect on the turn-on time of an FFS cell with α = 0. Moreover, the turn-off time is not dependent on the pretilt angle. Therefore, to enhance viewing angle characteristics, viewing angle compensation methods developed for a conventional FFS cell with α ≠ 0 can be directly applied to a zero-rubbing-angle cell with little modification^17^,^18^,^19^,^20^,^21^.

**Figure 8:**
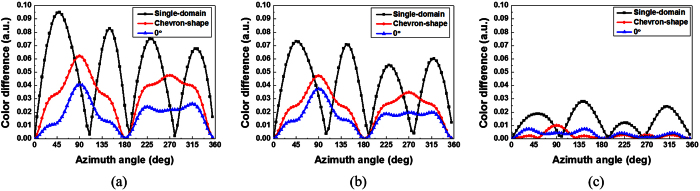
Dependence of the color shift on the azimuth angle for a polar angle of 60° at (**a**) 30%, (**b**) 60%, and (**c**) 100% of the maximum transmittance in a conventional single-domain FFS cell, a chevron-type FFS cell, and an FFS cell with α = 0. The chevron-shaped electrode has a bending angle of 10° (*W*: *L* = 2.8 μm: 6 μm).

In addition to the shorter response time, FFS cells with α = 0 exhibit a smaller off-axis color shift than do conventional single-domain FFS cells with α ≠ 0, as a result of the multi-domain effect induced by the virtual walls. To confirm the color characteristics of the FFS cells, we performed numerical calculations based on the extended 2 × 2 Jones matrix method^22^, using the commercial software package TechWiz LCD 3D. Figure 8 shows the dependence of the color shift on the viewing direction at 30%, 60%, and 100% of the maximum transmittance in a conventional single-domain FFS cell^4^, a chevron-type FFS cell^23^, and an FFS cell with α = 0. As an example, we calculated the color difference values as we varied the azimuth angle while maintaining a polar angle of 60°. In the conventional single-domain FFS cells, the LC molecules were rotated in one direction when an electric field was applied, thus causing the off-axis color shift to occur. To reduce the color shift, a chevron-type electrode structure has been widely used in which electric fields in two different directions are generated in each pixel, thus causing the LC molecules to rotate in two opposing directions^23^ and resulting in a smaller color shift than that in a single-domain electrode structure, as shown in Fig. 8. In the FFS cells with α = 0, the LC molecules are rotated in two opposing directions by the applied electric field, thereby compensating for the retardation that is dependent on the viewing direction without requiring the use of a chevron-type electrode structure. Therefore, FFS cells with α = 0 can effectively reduce the off-axis color shift by leveraging the same effect observed for the chevron-type electrode structure, as shown in Fig. 8.

## Experimental results

We fabricated FFS cells to verify our numerical results for the electro-optical characteristics of an FFS cell with α = 0. On the bottom substrate, transparent interdigitated and common electrodes were formed with an insulating layer between them. The width of the interdigitated electrodes was 2.8 μm, and the gap between them was either 4 μm or 6 μm. To achieve homogeneous alignment of the LC molecules, a thin polyimide layer was spin-coated onto each substrate and baked at 230 °C for 30 min. The polyimide layer was mechanically rubbed to align the LCs. The anti-parallel rubbing direction was set to 0° with respect to the interdigitated electrodes. The measured pretilt angle generated by the rubbing process was 2°. Then, the cell was assembled using silica spacers with a diameter of 3.5 μm. Finally, LCs were injected into the cell via capillary action. The parameters of the LC material used in the experiment were the same as those used in the numerical calculations. For comparison, conventional FFS cells with α = 10° were also fabricated with the same parameters as those used for the LC cells with α = 0.

**Figure 9:**
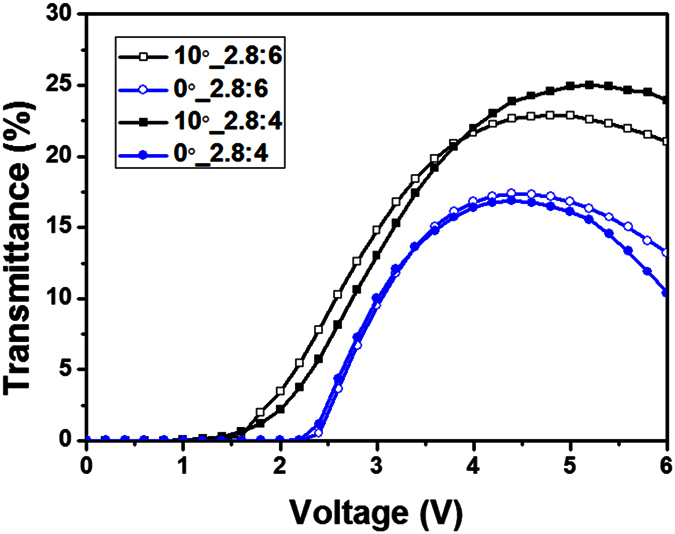
Measured voltage-transmittance curves of the fabricated FFS cells.

**Figure 10:**
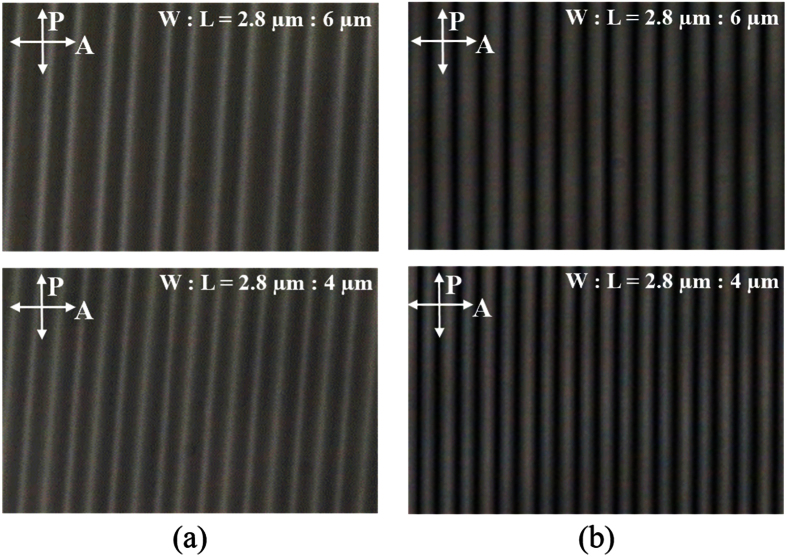
Polarizing optical microscope images of the bright state of the fabricated FFS cells. (**a**) α = 10°, (**b**) α = 0°.

To confirm the electro-optical characteristics of the fabricated LC cells, we measured their voltage-transmittance curves, as shown in Fig. 9. The experimental results demonstrated good correspondence with the calculated results. The FFS cells with α = 10° showed high brightness across the entire cell area, as shown in Fig. 10(a), whereas the FFS cells with α = 0 showed dark disclination lines, as shown in Fig. 10(b), which resulted in decreased transmittance. The transmittances of the conventional FFS cells with electrode gaps of 6 μm and 4 μm were 22.8% and 25.0%, respectively. In comparison, the transmittances of the FFS cells with α ≠ 0 were 17.4% and 16.9% for electrode gaps of 6 μm and 4 μm, respectively. Further study is needed to minimize the transmittance decrease by optimizing the LC material parameters, electrode structure, and cell gap.

**Figure 11:**
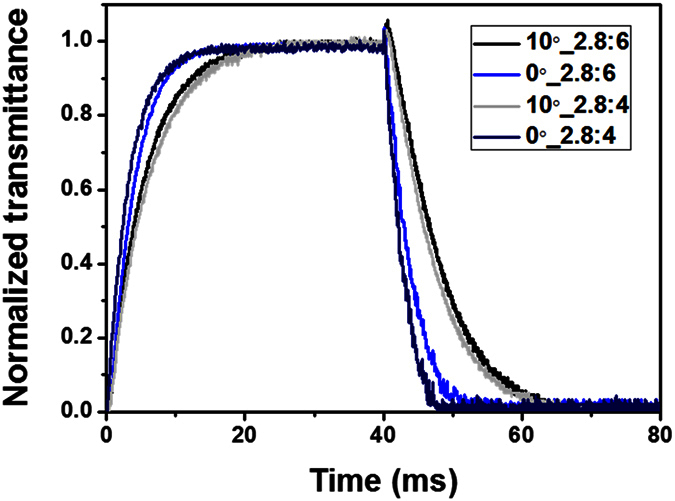
Measured switching behavior of the fabricated FFS cells.

We investigated the dynamic switching behavior of the fabricated LC cells, as shown in Fig. 11. To measure the response time of the fabricated LC cells, 4.8 V and 5 V were applied to the FFS cells with α = 10º and an electrode gap of 6 μm and 4 μm, respectively, whereas a voltage of 4.4 V was applied to the FFS cells with α = 0. When the width of the interdigitated electrodes and the gap between them were 2.8 μm and 6 μm, respectively, the turn-on and turn-off times of the FFS cell with α = 10º were 11.6 ms and 12.4 ms, respectively, whereas those of the FFS cell with α = 0 were 7.56 ms and 5.11 ms, respectively. The turn-off switching of the FFS cell with α = 0 was 2.5 times faster than that of the FFS cell with α = 10º. As the gap between the interdigitated electrodes decreased from 6 μm to 4 μm, the response time of the FFS cell with α = 0 was further reduced, whereas the response of the FFS cell with α = 10º was almost unchanged. When the width of the interdigitated electrodes and the gap between them were 2.8 μm and 4 μm, respectively, the turn-on and turn-off times of the FFS cell with α = 10º were 11.6 ms and 12.3 ms, respectively, whereas those of the FFS cell with α = 0 were 7.11 ms and 3.57 ms, respectively. The turn-off time of the FFS cell with α = 0 was 3.5 times shorter than that of the FFS cell with α = 10º.

Table 1 shows the turn-off times obtained from numerical calculations, the experimental results, and the predictions provided by the equation reported in ref. 16, respectively. Although the experimental results showed a deviation from both the calculated results and the predictions based on the equation reported in ref. 16, the response time decreased, as expected, as the pitch of the interdigitated electrodes was decreased. These results indicate that shorter response times can be achieved by simply using a rubbing angle of zero, without requiring additional fabrication steps or complicated drive schemes. The response time can be reduced by decreasing the pitch of the interdigitated electrodes as a result of the enhanced anchoring provided by the virtual walls. Notably, the electro-optical performance of the FFS cells with α = 0 exhibits trends very similar to those reported in ref. 16, which were obtained using a new IPS cell structure. The details of this new device structure have not yet been reported.

**Figure 12:**
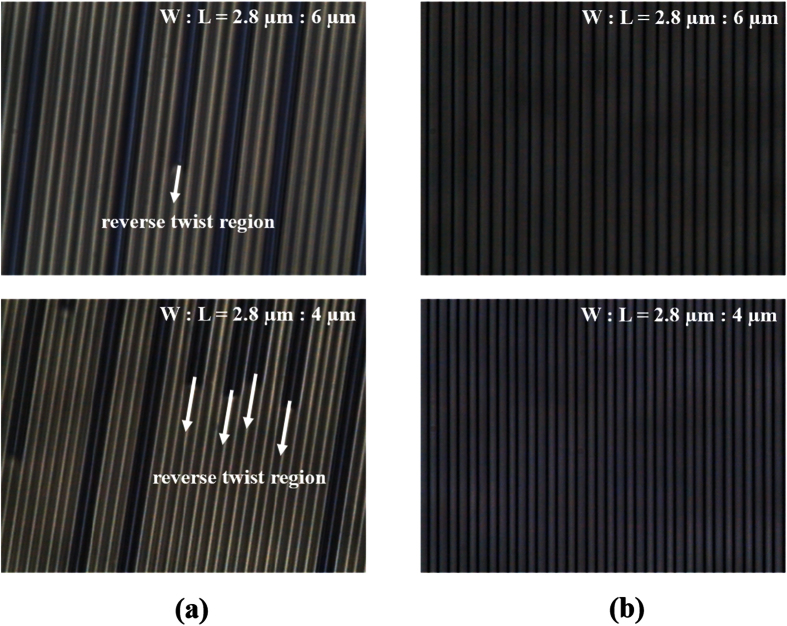
Images of FFS cells observed after a high voltage of 8 V was applied together with external pressure. (**a**) α = 10°, (**b**) α = 0°.

For this technology to be suitable for application in pressure-sensitive touch-based displays, it is necessary to obtain the external pressure resistance or the voltage-independent LC dynamic stability^24^,^25^,^26^. To confirm the dynamic stability of the LC molecules in the fabricated cells, we observed the LC texture with a polarized optical microscope, as shown in Fig. 12. In a conventional FFS cell with positive dielectric anisotropy, the reverse twist region was observed at the pixel edge, where the electric field direction differed from that of the active area. The reverse twist regions extended into the active region as the applied voltage was increased to 8 V. When an external pressure was applied to the LC cell, the reverse twist regions extended even further into the active region, as shown in Fig. 12(a).

To solve this problem, a bent pixel edge shape structure was used in an FFS panel to provide dynamic stability despite a corresponding reduction in optical efficiency^26^. In comparison, the FFS cells with α = 0 demonstrated excellent dynamic stability of its LC molecules, even when a high voltage or an external pressure was applied to the cell, as shown in Fig. 12(b). We believe that this phenomenon is caused by the virtual walls, which are stable and prevent the reverse twist generated at the pixel edges from moving into the active region.

## Conclusion

In summary, we demonstrated a fast FFS cell with a zero rubbing angle. The turn-on switching of an FFS cell with a zero rubbing angle is primarily governed by the dielectric torque, thus resulting in a short turn-on time. A short turn-off time was achieved through enhanced anchoring provided by virtual walls. This device does not require any additional fabrication steps or complicated drive schemes. Furthermore, this device exhibits a reduced color shift and excellent dynamic stability, even at a high applied voltage and under external pressure. We believe that this device may be a potential candidate for several applications requiring extremely fast switching of LCs, including automotive displays, digital signage, and field sequential color displays.

## Additional Information

**How to cite this article**: Choi, T.-H. *et al*. Fast fringe-field switching of a liquid crystal cell by two-dimensional confinement with virtual walls. *Sci. Rep.* **6**, 27936; doi: 10.1038/srep27936 (2016).

## Figures and Tables

**Table 1:** Turn-off times of FFS cells with α = 0 obtained through numerical calculations, experimental results, and predictions by the equation reported in ref. 16.

	Calculation	Experiment	Equation from ref. 16
W: L = 2.8 μm: 6 μm	6.12 ms	5.11 ms	4.52 ms
W: L = 2.8 μm: 4 μm	4.27 ms	3.57 ms	3.30 ms
